# Supplementary material for: Reward-related choices determine information timing and flow across macaque lateral prefrontal cortex
Source: Nat Commun. 2021 Feb 9;12:894. doi: 10.1038/s41467-021-20943-9 (PMC7873307; doi:10.1038/s41467-021-20943-9)
Supplement: Supplementary file 1 — Supplementary Information [file 41467_2021_20943_MOESM1_ESM.pdf]

Supplementary Information for

Reward-related choices determine information timing and flow across macaque lateral prefrontal cortex

Hua Tang, Ramon Bartolo, Bruno B. Averbeck*

Laboratory of Neuropsychology, National Institute of Mental Health, National Institutes of Health, Bethesda, MD 20892, USA.

Corresponding Author:

* Bruno B. Averbeck, PhD.

Email: averbeckbb@mail.nih.gov

Supplementary Text

Experimental Procedures

Local field potential (LFP) analysis

LFP recordings were low-pass filtered and sampled at 1 kHz. The signal was bandfiltered at 60 Hz with a narrow-band filter to eliminate the line noise; a sixth-order Butterworth filter was chosen to achieve a sharp drop-off in the stop-band, thus minimizing the effect of the filter on neighboring frequencies. We defined the following terminology to refer to particular frequency bands throughout the manuscript: delta range, 0-2 Hz; theta range2-6 Hz; alpha range, 6-10Hz; low beta range, 10-22 Hz; high beta range, 22-41 Hz; gamma range, 41-100 Hz.

The field potential data was first transformed into frequency space by computing the fast Fourier transform (FFT) of the signal on every electrode using a 256 ms window, stepped by 256 ms. We obtained the squared and log-transformed amplitude in a given source and target region for every electrode. Amplitude-amplitude coupling (AAC) was calculated for every electrode pair between each of the four regions in each hemisphere. We obtained estimates of coupling strength in two directions, rostral to caudal and caudal to rostral. Canonical correlation analysis (CCA) was used to compute the coupling. CCA computes coupling among multivariate observations in the source and target regions. More details can be found in our previous work [1].

We conducted our analyses using a Granger causal framework. Therefore, we first conducted CCA to estimate the effect of time-lagged activity on the current activity (i.e. effect of $Y_{(t-1)}$ on Y_t). This allows us to obtain an estimate of the coupling strength $\hat{Y}$ that could be inferred purely from the coupling of the signal at the target electrode onto itself. The first canonical value was used to obtain $\hat{Y}$, which was used to obtain the residual $\tilde{Y}$:

$$\tilde{Y}_t = Y_t - Y_{(t-1)}AB^T \qquad (1)$$

Here Y_t represents power across frequencies at time t and $Y_{(t-1)}$ represents power at each frequency in the previous time bin, both in the target region. The variables A and B represents the first columns of the canonical coefficient matrix that make up the first canonical variable for correlations between $Y_{(t-1)}$ and Y_t.

We then computed cross-frequency power coupling across the four regions in the same hemisphere by regressing the amplitude component of the signal from the source region (X_t) on the amplitude component of the residual signal in the target region (residual $\tilde{Y}$). More explicitly, we calculated instantaneous coupling in 256 ms windows between the source, X_t, and the target, $\tilde{Y}_t$, regions for each electrode pair. Cross frequency power coupling was calculated in the CCA framework across all trials, simultaneously across all frequency bands. The final cross-frequency coupling matrix P was calculated with:

$$P = BA^T, \qquad (2)$$

Here A and B represents the first columns of the canonical coefficient that make up the first canonical variable for X_t and residual $\tilde{Y}_t$.

Paired t-tests were performed to establish the statistical significance of the difference between rostral-to-caudal and caudal-to-rostral coupling. This was done separately for each frequency pair, by testing whether the mean of the difference between rostral-to-caudal and caudal-to-rostral coupling was significantly different from zero. To assess the information flow of frequency coupling, 1-way ANOVA was used to compare the interaction of region pairs with different ordinal distances.

Supplementary Results

LFP analysis.

To examine whether there was directed information flow in the LFPs, we analyzed the LFPs recorded simultaneously from the four Utah arrays while the animals performed the task. We computed cross-spectral power coupling for every electrode pair across the four regions in each hemisphere. We first decomposed the signal into its spectra using the short-time FFT and then obtained the amplitude at each frequency in each time window. Then, canonical-correlation analysis (CCA) was used to compute the cross-frequency coupling (CFC). We first removed the effect of the previous time bin in the target region by computing the CCA between the current time window and the previous time window, and then using the first canonical component to compute a prediction. We then obtained the residual power after removing this prediction. We then predicted the residual activity in the target electrode, using an electrode from a different array, at zero time lag, as we have done previously [1].

CFC was calculated across the frequency spectrum for every electrode pair across the four arrays. As with the spike data analysis, the coupling strength was also estimated in two directions, rostral-to-caudal and caudal-to-rostral. Rostral-to-caudal coupling was defined as coupling in which the source electrode came from a region rostral to the target electrode, and vice versa for caudal-to-rostral coupling. For example, the coupling between the amplitude in the rdlPFC and cdlPFC is defined as rostral-to-caudal. Results are presented collapsed across all cross-regional pairs (Fig. S13**a**-**b**), as well as separately for every cross-regional pair (Fig. S14). In all cases, these are averaged across all electrode pairs and the two animals.

We first examined CFC across the four regions. Both rostral-to-caudal and caudal-to-rostral coupling was strongest between low frequencies, especially theta, alpha, and low beta frequencies (Fig. S13**a**-**b**). Examining differences in coupling strength between the two directions (Fig. S13**c**) showed dominant rostral-to-caudal coupling between low frequencies (theta, alpha and low beta ranges to alpha and low beta ranges). Caudal-to-rostral coupling was dominant between the low-frequency range (theta and alpha ranges) and the high-frequency range (gamma range).

Next, we analyzed differences between rostral-to-caudal and caudal-to-rostral directions across the six cross-regional pairs (Fig. S14). It revealed that the rostral-to-caudal coupling of theta and alpha amplitude to alpha and low beta amplitude was consistent across the six pairs. Interactions between vlPFC and mdlPFC/cdlPFC also showed dominant rostral-to-caudal coupling of low beta amplitude to high beta amplitude and high beta amplitude to gamma amplitude (Fig. S14**e**-**f**), whereas these asymmetries were largely absent among interactions between the rdlPFC and the other regions (Fig. S14**a**-**c**). The caudal-to-rostral coupling existed across all the six cross-regional pairs but was most dominant between the rdlPFC and the other three regions (Fig. S14**a**-**c**).

To examine cross-regional patterns in coupling across the rostro-caudal axis more closely, we compared the coupling strength between different frequency ranges at different ordinal distances as we did for the spikes. These analyses were also split by block types (Fig. S13**d**-**i**). An example with the source frequency range as alpha is shown in Fig. S13**d**-**e**, the coupling strength between frequencies in the source region and other frequencies in the target region were plotted as continuous lines, split by the ordinal distances between the source and target regions. Next, we examined the mean coupling strength for each frequency range in the target region. We found directed information flow, similar to what we found in the spike data.

There was stronger flow in the caudo-rostral direction than in the rostro-caudal direction in the coupling between theta/alpha and gamma frequencies (Fig. S13**f**, unpaired t-test, $t(94) = 6.51$, $p < 0.001$). There was stronger flow in the rostro-caudal direction than in the caudo-rostral direction for the coupling between theta/alpha and low beta (Fig. S13**g**, unpaired t-test, $t(94) = 3.54$, $p < 0.001$), low beta and high beta (Fig. S13**h**, unpaired t-test, $t(94) = 3.70$, $p < 0.001$), high beta and gamma (Fig. S13**i**, unpaired t-test, $t(94) = 2.75$, $p < 0.01$) frequency ranges. But there was no significant difference found between What and Where blocks for any of these conditions.

Taken together, these results revealed that the coupling between subregions of the LPFC also showed directed flows in different frequency ranges. But, since we did not extract the object and action information from the LFPs, as did for the spike data analysis. It is not clear whether these directed flows of cross-frequency coupling are related to the transformation/processing of any specific domain of information.

Supplementary Figures and Tables

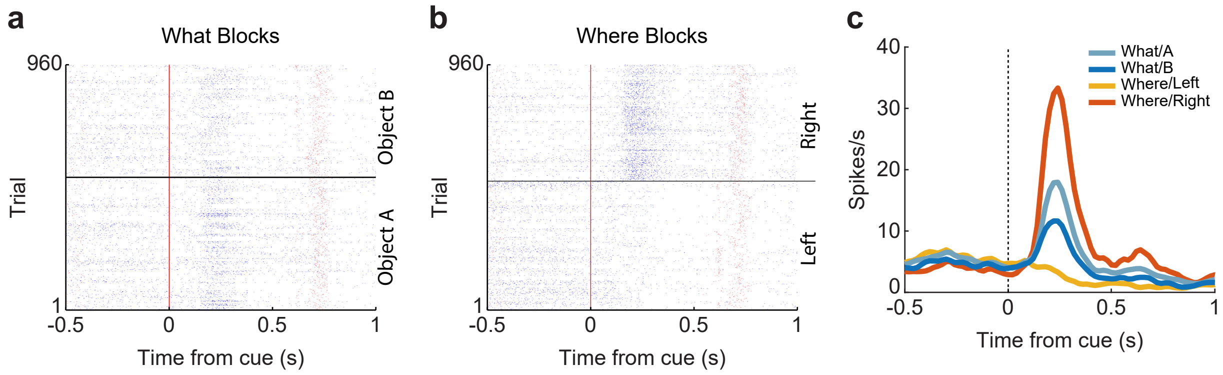

Fig. S1. Neural responses of an example neuron. (**a-b**) Raster plots of an example neuron in What (**a**) and Where (**b**) blocks. Each row represents the spikes during a trial. Red dots along each line represent fixation, cue onset, and outcome time. Since the object varies in each block, trials were sorted by preferred (object A) and non-preferred (object B) objects in What blocks. In Where blocks, trials were sorted by chosen actions (left/right). (**c**) Average activity for the example neuron in each option and block type combination. Source data are provided as a Source Data file.

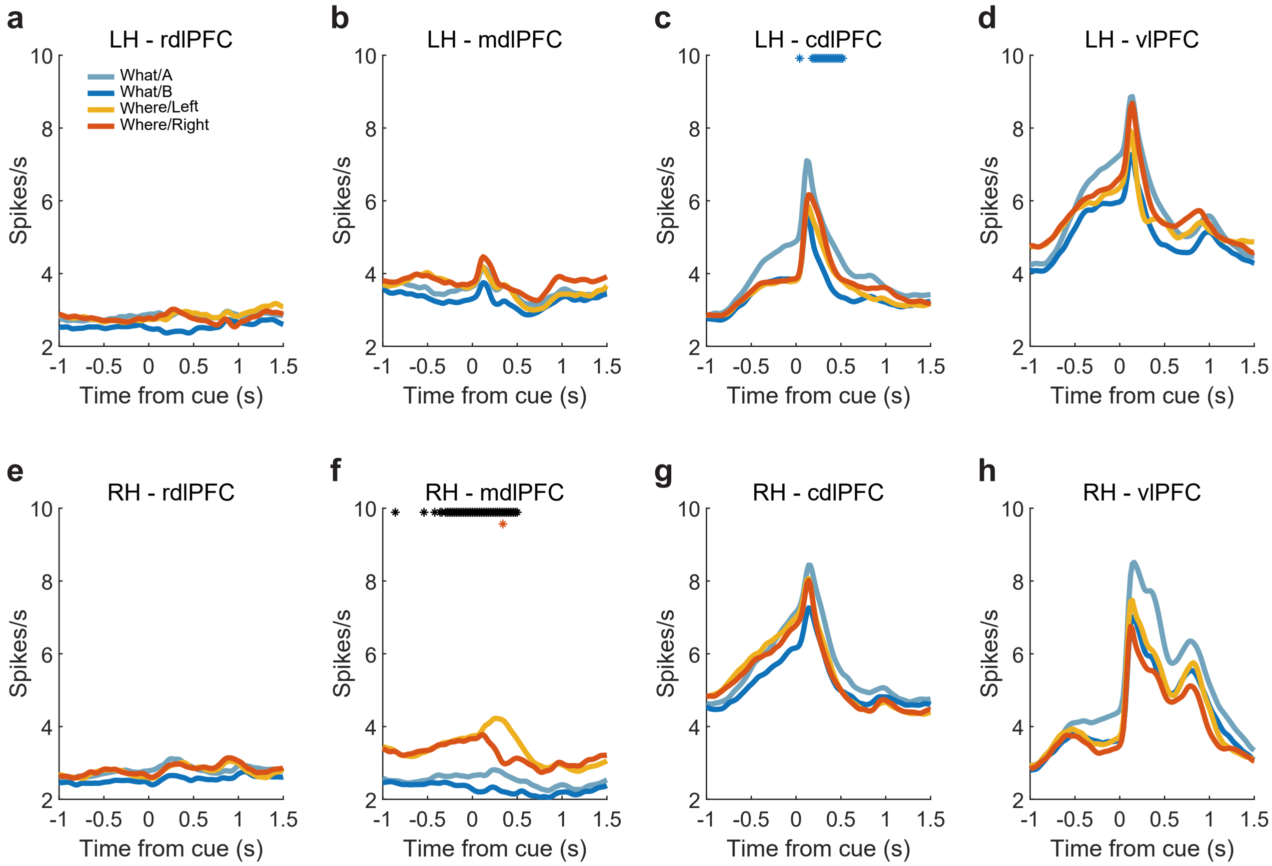

Fig. S2. Population activity of responsive neurons in each region. (**a-h**) A sliding-window ANOVA was performed on spikes counted in 50 ms bins, advanced in 10 ms increments. Neural responses were calculated for all neurons showing a significant effect in at least five continual windows from 0 to 1200 ms after cue onset. Trials were sorted by preferred (object A) and non-preferred (object B) objects in What blocks; sorted by chosen actions (left/right) in Where blocks. The black * symbols at the top of the panel indicate a significant difference among the four conditions (1-way ANOVA, $p < 0.05$). The colored * symbols at the top of some panels indicate a significant difference (two-sided unpaired t-test, $p < 0.05$) between two options in each block type (blue for What blocks, orange for Where blocks). LH/RH indicates the left/right hemisphere. Source data are provided as a Source Data file.

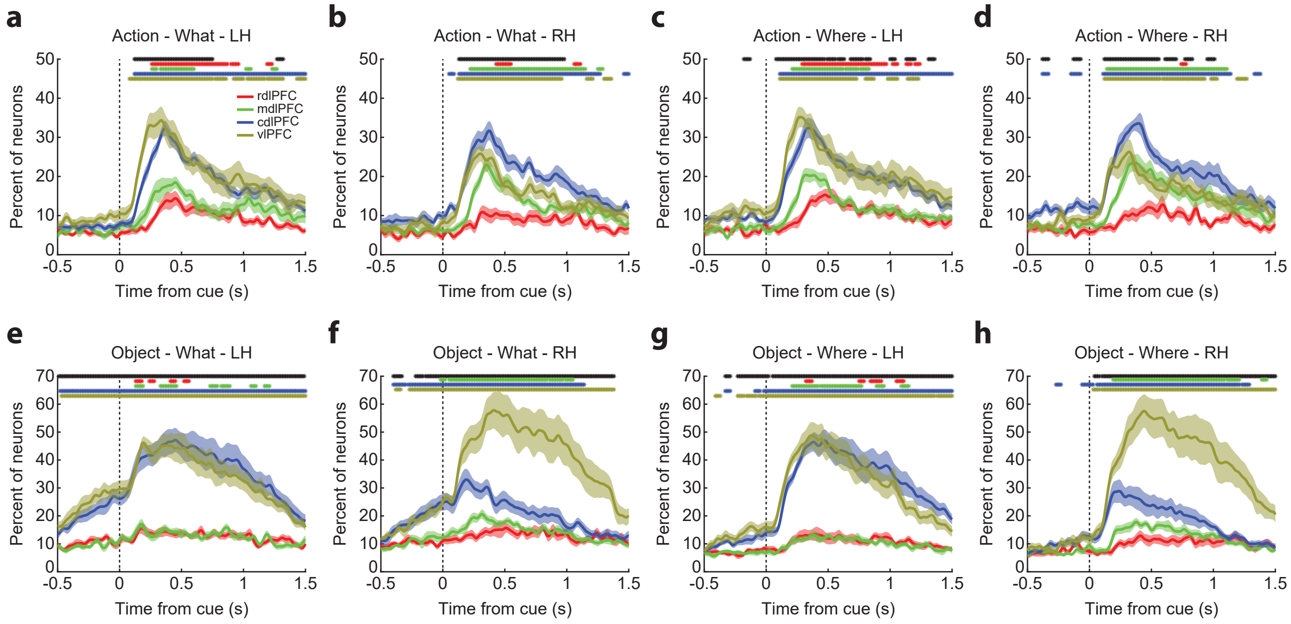

Fig. S3. Population encoding of chosen action and chosen object, split by cerebral hemisphere. (**a-h**) Percentage of task-related neurons in each array that encoded action in What (**a-b**) or Where blocks (**c-d**), or encoded object in What (**e-f**) or Where (**g-h**) blocks. LH/RH indicates the left/right hemisphere. Shaded zones represent mean ± SEM, n = 8 sessions. The black * symbols at the top of each panel indicate a significant difference among the four regions (1-way ANOVA, $p < 0.01$). The colored * symbols indicate a significant difference (two-sided paired t-test, $p < 0.01$) of task-related neuron percentage between the corresponding region and its baseline. Source data are provided as a Source Data file.

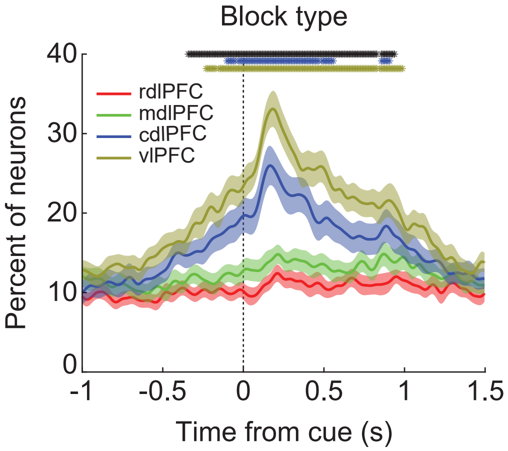

Fig. S4. Population encoding of block type. Percentage of task-related neurons in each region that encoded block types. Shaded zones represent mean ± SEM, n = 8 sessions. The black * symbols at the top indicate a significant difference among the four regions (1-way ANOVA, $p < 0.01$). The colored * symbols indicate a significant difference (two-sided paired t-test, $p < 0.01$) of task-related neuron percentage between the corresponding region and its baseline. Source data are provided as a Source Data file.

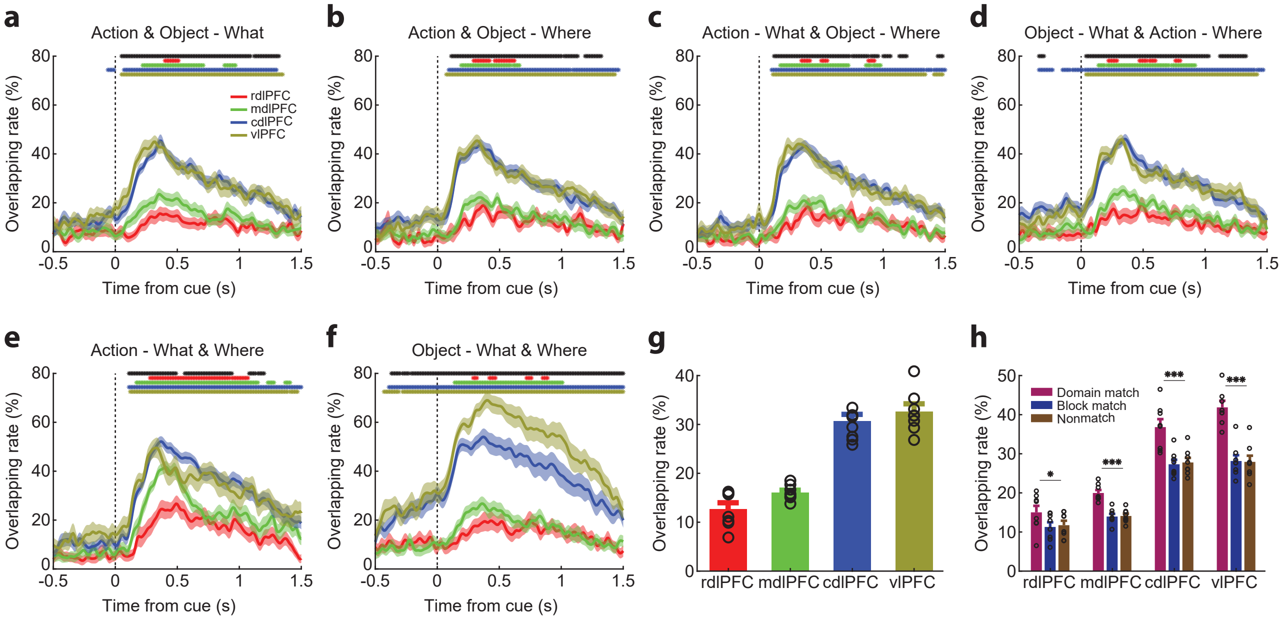

Fig. S5. Neuronal population overlap between different task factors. (**a**-**f**) The overlapping rate of task-related neurons in each region that encoded the chosen action and chosen object in What blocks (**a**), action and object in Where blocks (**b**), action in What and Where blocks (**c**), action and object in Where blocks (**d**), action in What blocks and object in Where blocks (**e**), object in What blocks and action in Where blocks (**f**). (**g**-**h**) The overlap rate of task-related neurons in each region (**g**), split by domain/block type (**h**), averaged from 0 to 1.5 seconds from cue onset. Shaded zones and error bars represent mean ± SEM, n = 8 sessions for each bar or box. 1-way ANOVA was used to compare multi-population, *$p < 0.05$, **$p < 0.01$, ***$p < 0.001$. The black * symbols at the top of each panel indicate a significant difference among the four regions (1-way ANOVA, $p < 0.01$). The colored * symbols indicate a significant difference (two-sided paired t-test, $p < 0.01$) of task-related neuron overlapping rate between the corresponding region and its baseline. Source data are provided as a Source Data file.

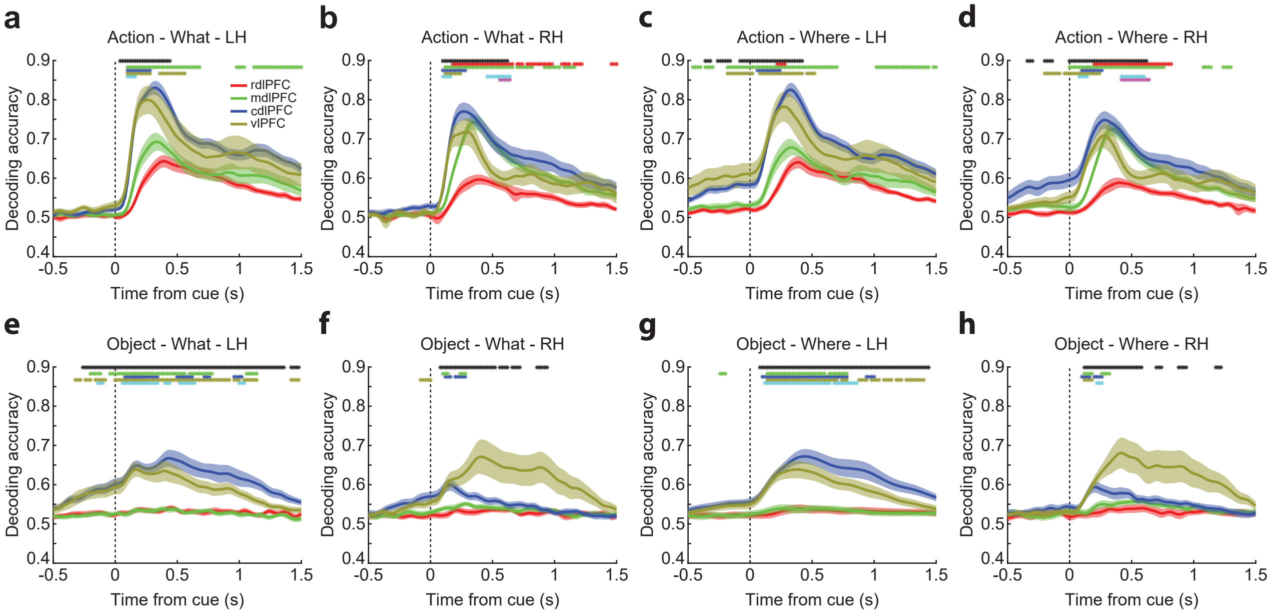

Fig. S6. Decoding of chosen action and chosen object, split by cerebral hemisphere. The time course of decoding accuracy in each region for encoding action in What blocks (**a-b**) or Where blocks (**c-d**), objects in What (**e-f**) or Where blocks (**g-h**). LH/RH indicates the left/right hemisphere. Shaded zones represent mean ± SEM, n = 8 sessions. The black * symbols at the top of each panel indicate a significant difference among the four regions (1-way ANOVA, $p < 0.01$). The red, green, blue, yellow, cyan and magenta * symbols indicate a significant difference (two-sided paired t-test, $p < 0.01$) between the rdlPFC and mdlPFC, rdlPFC and cdlPFC, rdlPFC and vlPFC, mdlPFC and cdlPFC, mdlPFC and vlPFC, cdlPFC and vlPFC. Source data are provided as a Source Data file.

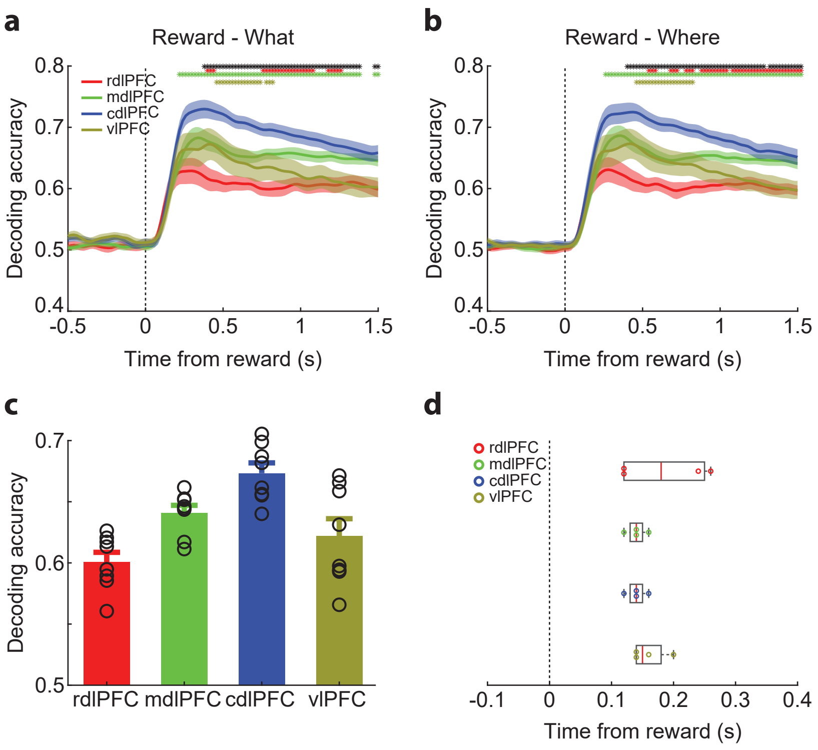

Fig. S7. Decoding of reward. (**a-b**) The time course of decoding accuracy in each region that encoding reward in What blocks (**a**) or Where blocks (**b**). (**c**) The decoding accuracy in each region, averaged from 0 to 1.5 seconds from cue onset. (**d**) The response latency of the neuronal populations in each region. The hollow circles represent the response latency of each condition, n = 4 conditions. Boxplot box indicates first and third quartile, the center line of the box indicates the median, and whisker lengths reflect the inter-quartile range multiplied by 1.5. Shaded zones and error bars represent mean ± SEM, n = 8 sessions for each bar or box. The black * symbols at the top of each panel indicate a significant difference among the four regions (1-way ANOVA, $p < 0.01$). The red, green, blue, yellow, cyan and magenta * symbols indicate a significant difference (two-sided paired t-test, $p < 0.01$) between the rdlPFC and mdlPFC, rdlPFC and cdlPFC, rdlPFC and vlPFC, mdlPFC and cdlPFC, mdlPFC and vlPFC, cdlPFC and vlPFC. Source data are provided as a Source Data file.

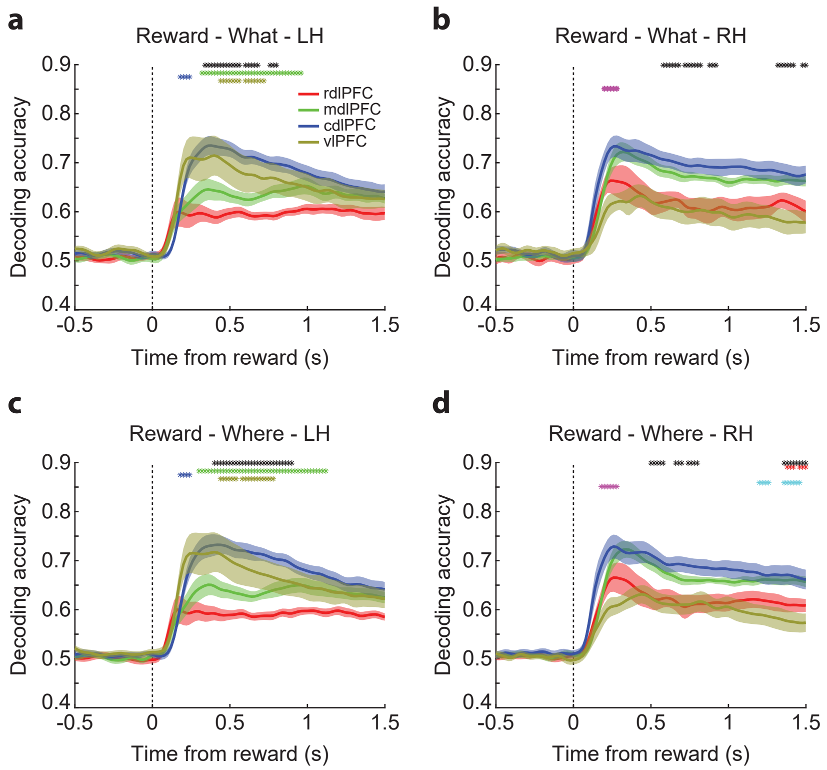

Fig. S8. Decoding of reward, split by cerebral hemisphere. (**a-d**) The time course of decoding accuracy in each region that encoding reward in What blocks (**a-b**) or Where blocks (**c-d**). Shaded zones represent mean ± SEM, n = 8 sessions. The black * symbols at the top of each panel indicate a significant difference among the four regions (1-way ANOVA, $p < 0.01$). The red, green, blue, yellow, cyan and magenta * symbols indicate a significant difference (two-sided paired t-test, $p < 0.01$) between the rdlPFC and mdlPFC, rdlPFC and cdlPFC, rdlPFC and vlPFC, mdlPFC and cdlPFC, mdlPFC and vlPFC, cdlPFC and vlPFC. Source data are provided as a Source Data file.

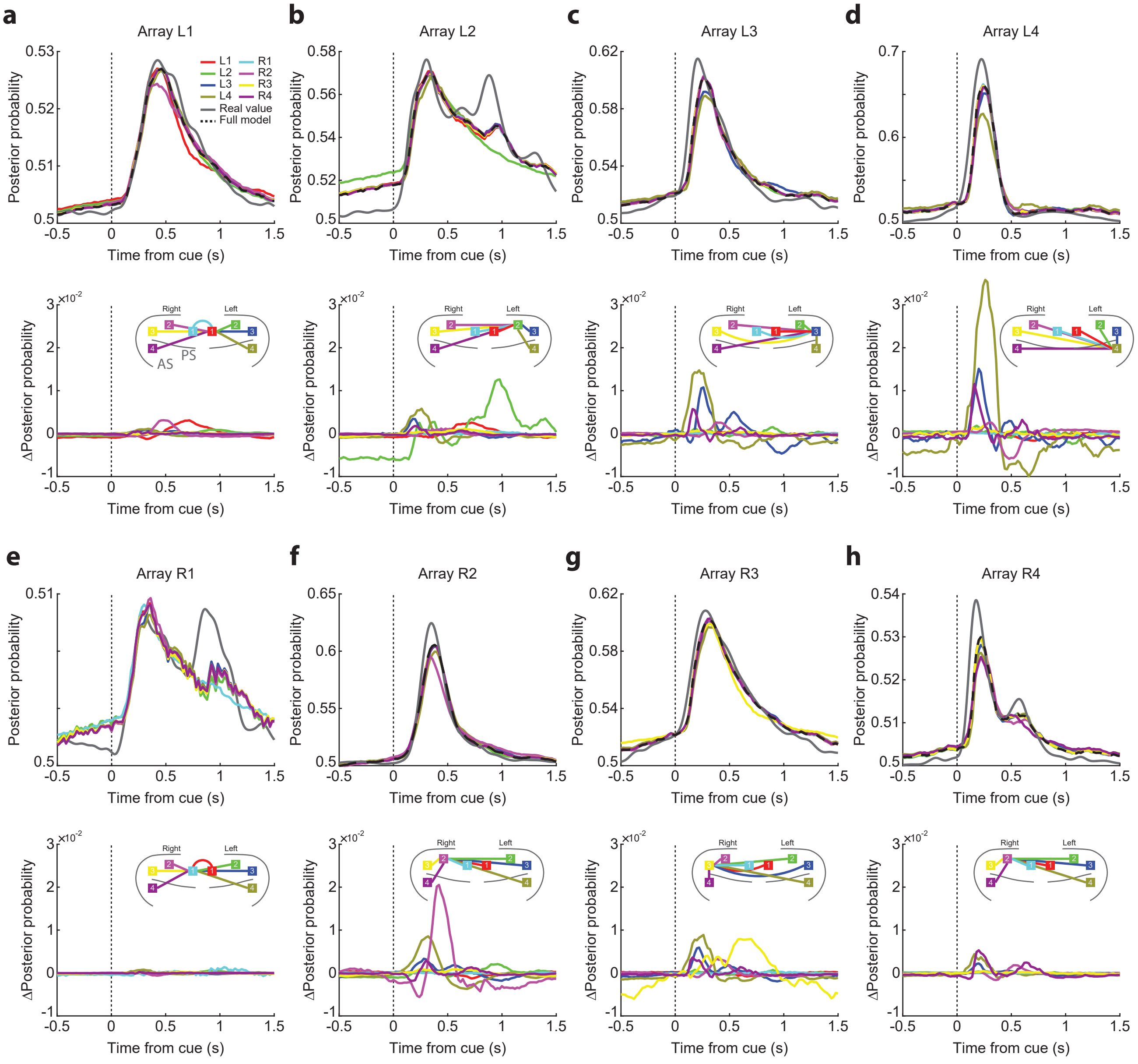

Fig. S9. Information transfer model extended. Same data as in Fig. 5, without averaging across hemispheres. An example of the posterior probability of the information transfer model, predicting the decoding accuracy of action in Where blocks of the array L1 (**a**), L2 (**b**), L3 (**c**), L4 (**d**), R1 (**e**), R2 (**f**), R3 (**g**) and R4 (**h**) with the input from all eight arrays. (**a-h**, top) The posterior probability of the raw value, the Full model and the Partial models. (**a-h**, bottom) The difference of posterior probability between the Partial models and the Full model. Inset: the colored lines connect the dropped input arrays (indicated by the color of the line) and the output array. Source data are provided as a Source Data file.

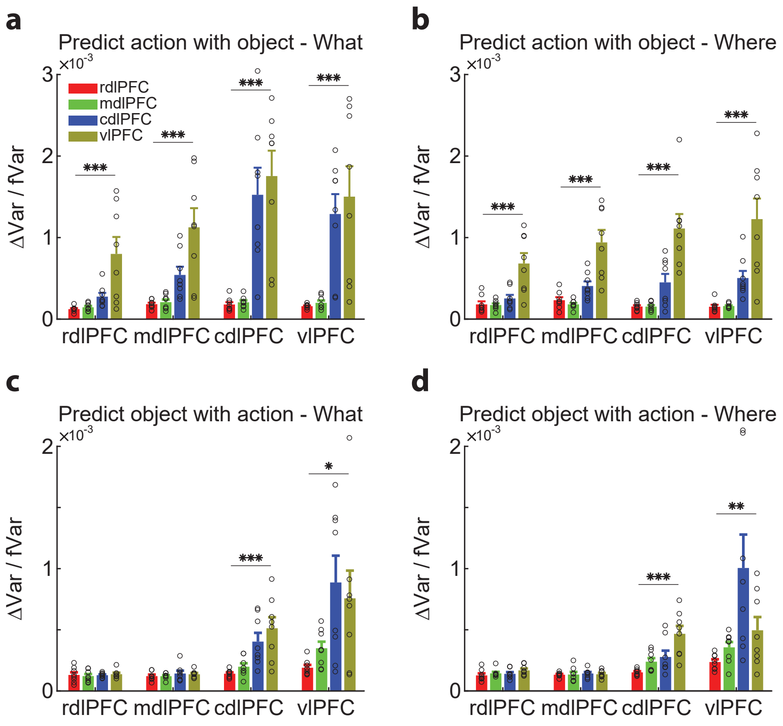

Fig. S10. Prediction variance across regions. The difference of variance between the Partial models and the Full model, when predicting decoding accuracy of action with object information in What and Where blocks (**a-b**), predicting decoding accuracy of the object with the action information in What and Where blocks (**c-d**). Legends indicate input regions, and the x-axis indicates output regions. Effects of the regions at corresponding locations in the left and right hemispheres have been averaged. Error bars represent mean ± SEM, n = 8 sessions for each bar. 1-way ANOVA was used to compare multi-population, *p < 0.05, **p < 0.01, ***p < 0.001. Source data are provided as a Source Data file.

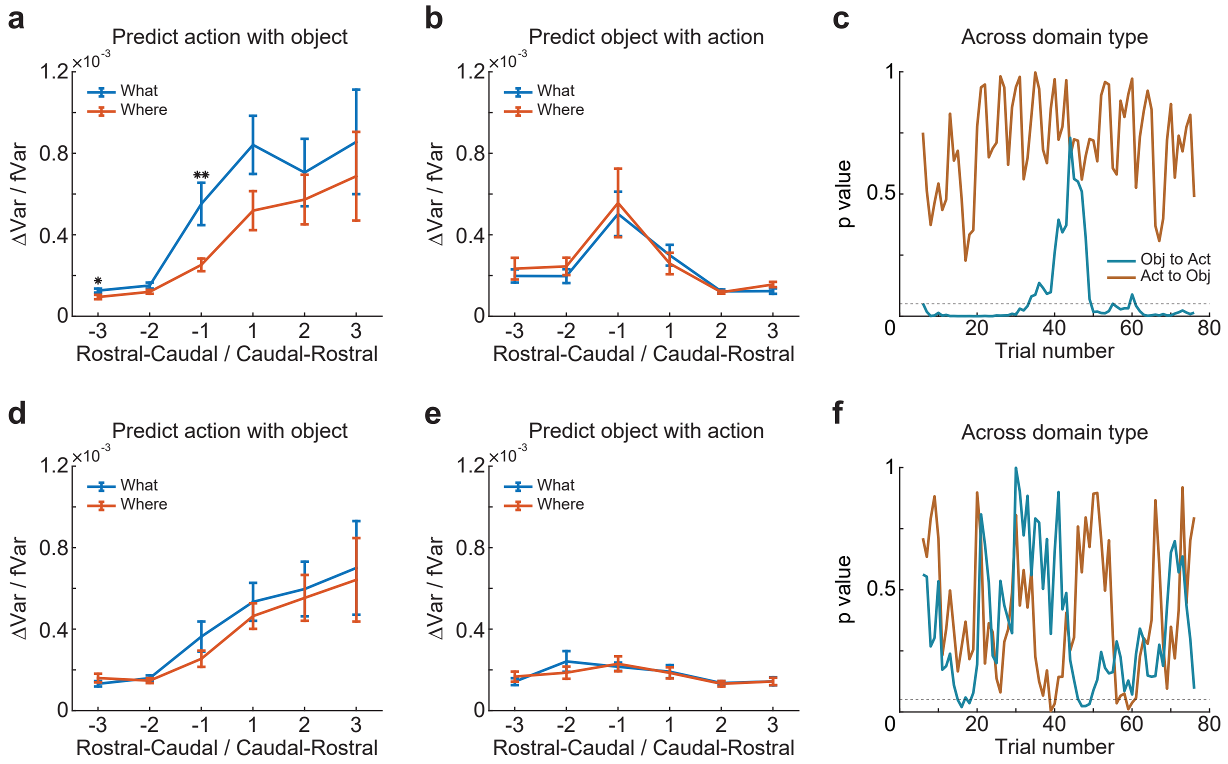

Fig. S11. Prediction of chosen action and chosen object across domain type plotted by individual monkeys. (**a-b**, **d-e**) The difference of variance between the Partial models and the Full model, plotted as a function of the ordinal distance between the input and output regions, predicting decoding accuracy of action with object (**a**, **d**) and predicting object with action (**b**, **e**). Error bars represent mean ± SEM, n = 16/32/48/48/32/16 for the ordinal distance of -3/-2/-1/1/2/3. A two-sided t-test was used to compare two populations, *p = 0.0255, **p = 0.0073. (**c**, **f**) The p-value of the difference in information flow between tasks (What and Where), aligned by the trial index. Bin = 10 trials, step = 1 trial. Dash lines represent p = 0.05, "Act" represents action, "Obj" represents object. (**a-c**) Data from monkey V. (**d-f**) Data from monkey W. Source data are provided as a Source Data file.

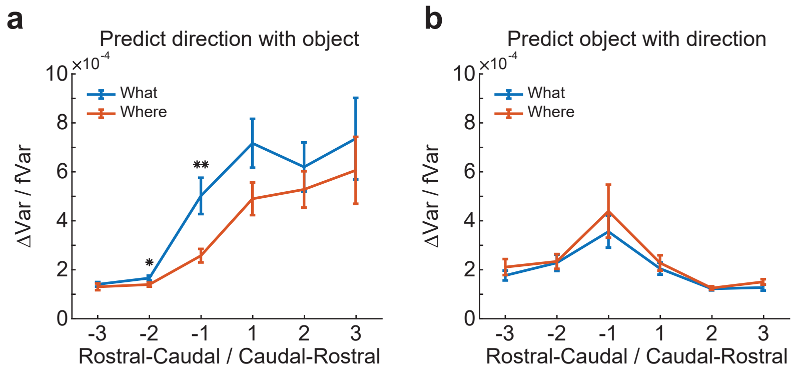

Fig. S12. Prediction of chosen action and chosen object, using only lagged values. The difference of variance between the Partial models and the Full model, plotted as a function of the ordinal distance between the input and output arrays/regions, predicting action with object (**a**) and predicting object with action (**b**). Error bars represent mean ± SEM, n = 32/64/96/96/64/32 for the ordinal distance of -3/-2/-1/1/2/3. A two-sided t-test was used to compare two populations, *p = 0.0451, **p = 0.0023. Source data are provided as a Source Data file.

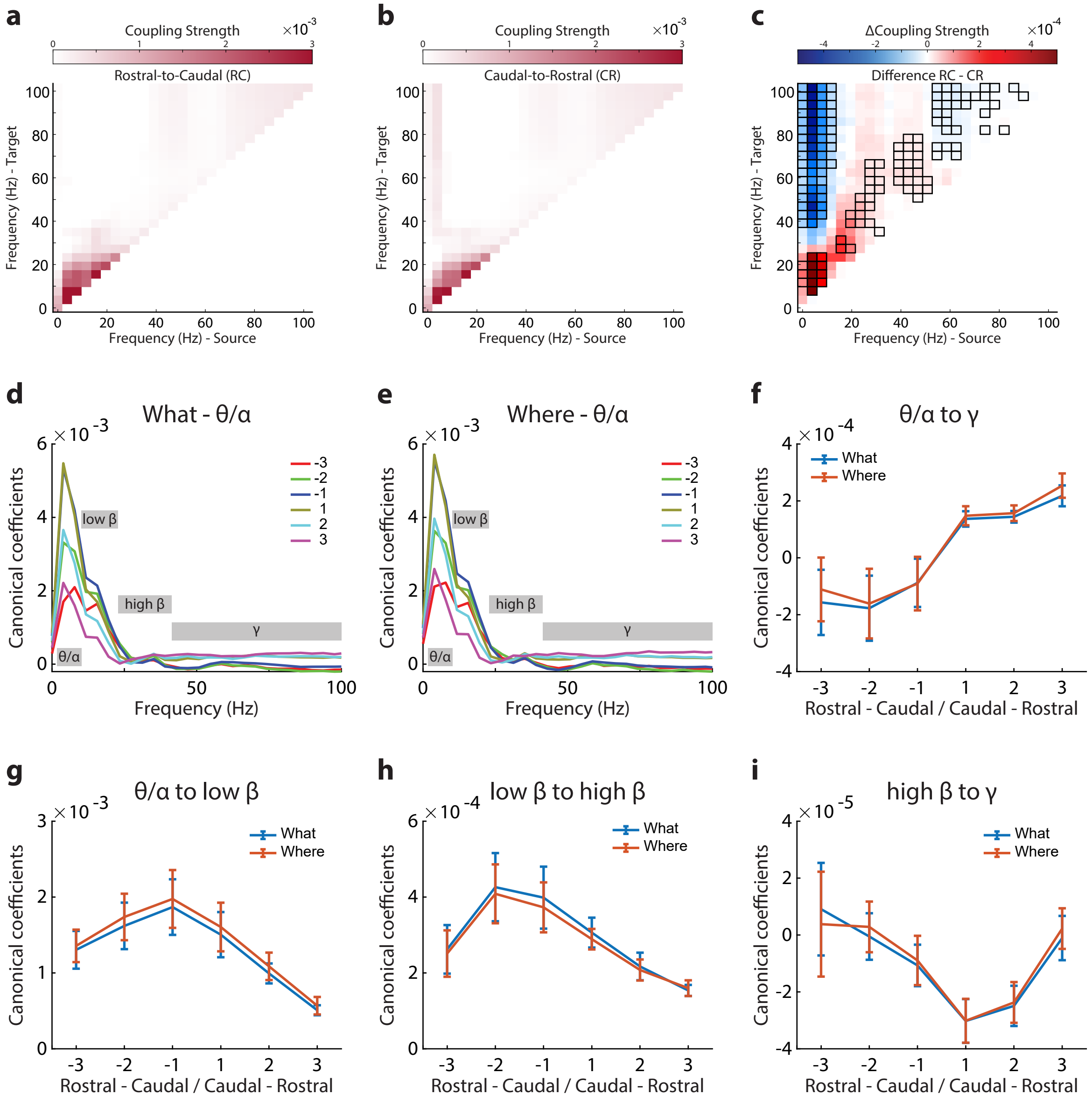

Fig. S13. Rostral-to-caudal and caudal-to-rostral cross frequency power coupling. (**a-b**) Cross frequency power coupling (CFC) in the rostral-to-caudal (**a**) and caudal-to-rostral (**b**) directions. Color bars indicate the canonical correlation-derived coupling coefficients. (**c**) Difference between rostral-to-caudal and caudal-to-rostral in CFC strength. Significant differences are marked by black outlines (two-sided unpaired t-test, $p < 0.05$). Results are depicted averaged across all electrodes, cross-regional pairs, and animals. (**d-e**) Averaged cross-frequency coupling with frequency range in source region as theta/alpha. The coupling strength between frequencies in the source region and other frequencies (indicated by gray bars) in the target region are plotted as continuous lines, split by the ordinal distances between the source and target regions. (**f-i**) Cross-frequency coupling strength plotted as a function of the ordinal distance between the source and target regions, when the frequency pairs in the source and target region are gamma and theta/alpha (**f**), theta/alpha and low beta (**g**), low beta and high beta (**h**), high beta and gamma (**i**). Other frequency range pairs without significant differences between these two directions are not shown here. Error bars represent mean ± SEM, n = 32/64/96/96/64/32 for the ordinal distance of -3/-2/-1/1/2/3. Source data are provided as a Source Data file.

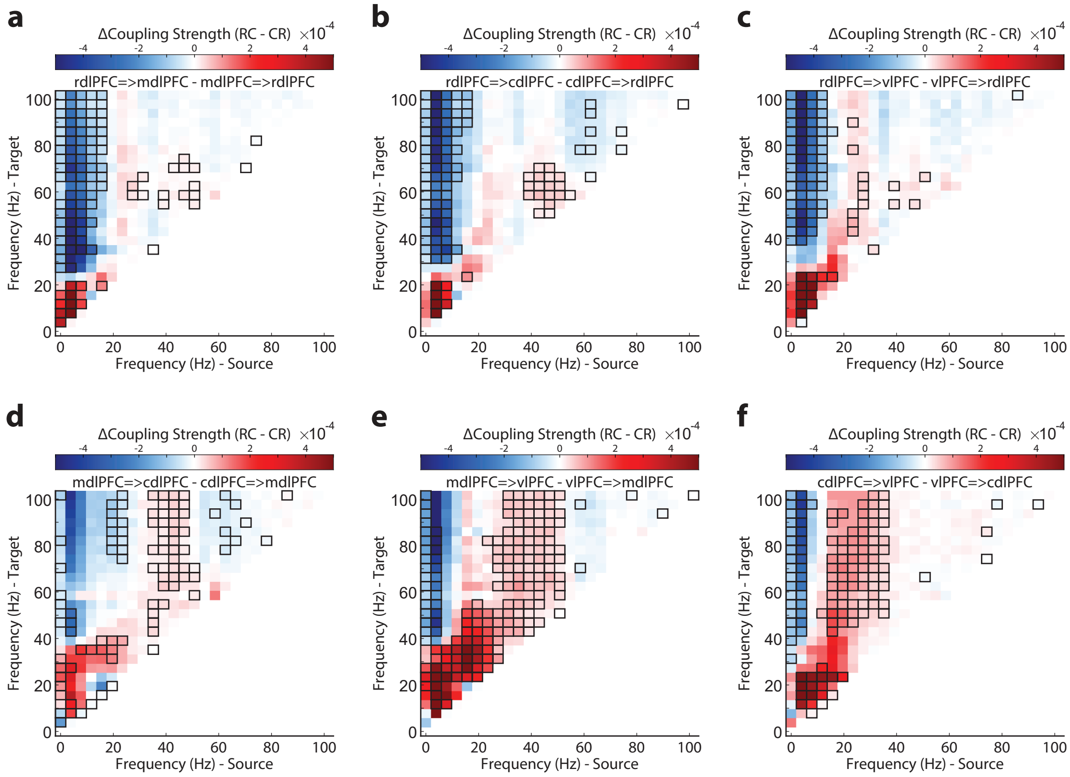

Fig. S14. Rostral-to-caudal and caudal-to-rostral amplitude coupling. (**a**-**f**) Difference in rostral-to-caudal and caudal-to-rostral AAC strength for cross-frequency coupling between rdlPFC and mdlPFC (**a**), rdlPFC and cdlPFC (**b**), rdlPFC and vlPFC (**c**), mdlPFC and cdlPFC (**d**), mdlPFC and vlPFC (**e**), cdlPFC and vlPFC (**f**). Significant differences are marked by black outlines (two-sided unpaired t-test, $p < 0.05$). Source data are provided as a Source Data file.

Table S1. The number of neurons recorded in each array for each session.

Session	Left hemisphere				Right hemisphere			
	rdlPFC	mdlPFC	cdlPFC	vlPFC	rdlPFC	mdlPFC	cdlPFC	vlPFC
w20160112	81	82	129	57	88	122	85	36
w20160113	111	105	157	69	73	95	97	40
w20160121	95	100	127	32	67	103	93	60
w20160122	85	83	110	28	48	104	78	49
v20160929	81	49	140	118	163	123	74	129
v20160930	118	107	142	123	130	122	81	119
v20161005	114	128	126	139	146	108	139	126
v20161017	88	64	102	113	19	87	96	29

Table S2. The number of responsive neurons recorded in each array.

Block type	Left hemisphere				Right hemisphere			
	rdlPFC	mdlPFC	cdlPFC	vlPFC	rdlPFC	mdlPFC	cdlPFC	vlPFC
What	125	88	206	173	108	97	129	100
Where	73	72	130	87	72	97	121	71

Table S3. The response latency of the neuronal population encoding action and object*.

Domain type	Block type	Left hemisphere				Right hemisphere			
		rdlPFC	mdlPFC	cdlPFC	vlPFC	rdlPFC	mdlPFC	cdlPFC	vlPFC
Action	What	0.26 s	0.26 s	0.12 s	0.08 s	0.29 s	0.22 s	0.05 s	0.13 s
	Where	0.29 s	0.21 s	0.11 s	0.11 s	0.73 s	0.14 s	-0.15 s	0.12 s
Object	What	0.13 s	0.12 s	-0.48 s	-0.47 s	nan	0.05 s	-0.4 s	-0.38 s
	Where	0.84 s	0.21 s	-0.38 s	-0.41 s	nan	0.19 s	-0.06 s	0.04 s

* Aligned to cue onset.

Table S4. The response latency of the neuronal population encoding reward[*].

Block type	Left hemisphere				Right hemisphere			
	rdlPFC	mdlPFC	cdlPFC	vlPFC	rdlPFC	mdlPFC	cdlPFC	vlPFC
What	0.26 s	0.14 s	0.16 s	0.14 s	0.18 s	0.14 s	0.14 s	0.2 s
Where	0.24 s	0.16 s	0.14 s	0.14 s	0.12 s	0.12 s	0.14 s	0.16 s

* Aligned to choice outcome.

SI References

1. Marton CD, Fukushima M, Camalier CR, Schultz SR, Averbeck BB. Signature Patterns for Top-Down and Bottom-Up Information Processing via Cross-Frequency Coupling in Macaque Auditory Cortex. *eNeuro* **6**, (2019).
