# Supplementary figures and images for: Reward-related choices determine information timing and flow across macaque lateral prefrontal cortex
Source: Nat Commun. 2021 Feb 9;12:894. doi: 10.1038/s41467-021-20943-9 (PMC7873307; doi:10.1038/s41467-021-20943-9)

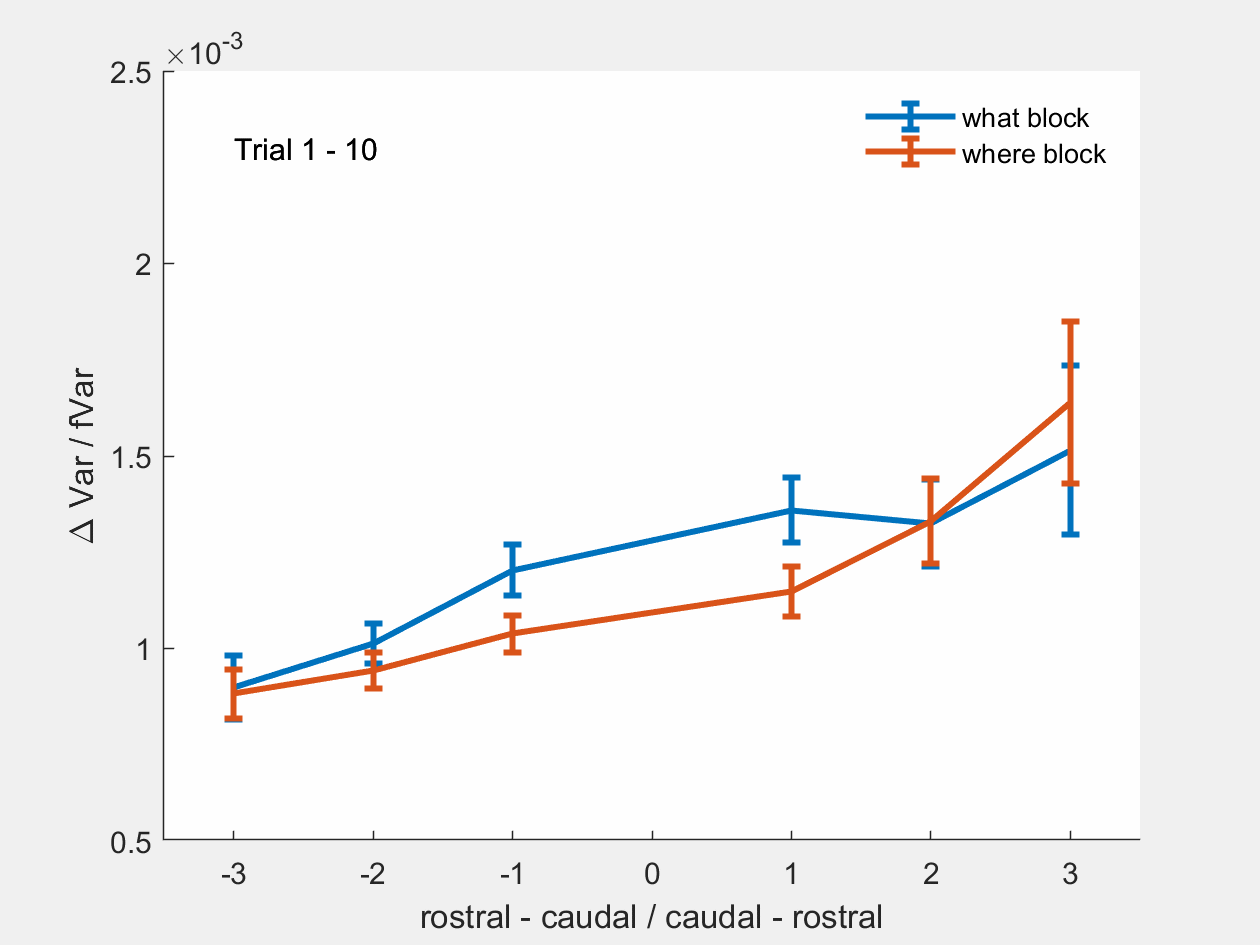

Supplement: Supplementary file 3 — Supplementary Movie 1 [file 41467_2021_20943_MOESM3_ESM.gif]
