# Supplementary material for: Reward-related choices determine information timing and flow across macaque lateral prefrontal cortex
Source: Nat Commun. 2021 Feb 9;12:894. doi: 10.1038/s41467-021-20943-9 (PMC7873307; doi:10.1038/s41467-021-20943-9)
Supplement: Supplementary file 4 — Description of additional supplementary files [file 41467_2021_20943_MOESM4_ESM.docx]

Description of additional supplementary information

Title: Supplementary Movie 1. Predict action with object identity across trials.

Description: The difference of variance between the Partial models and the Full model, grouped by trial index in each block, plotted as a function of the distance between the input and output regions, predicting the decoding accuracy of action with the object. Bin = 10 trials, step = 1 trial. Error bars represent mean ± SEM, n = 32/64/96/96/64/32 for the ordinal distance of -3/-2/-1/1/2/3. A two-sided t-test was used to compare two populations, *p < 0.05, **p < 0.01, ***p < 0.001.
